# Tolerance: the forgotten child of plant resistance

**DOI:** 10.7717/peerj.3934

**Published:** 2017-10-16

**Authors:** Robert K.D. Peterson, Andrea C. Varella, Leon G. Higley

**Affiliations:** 1 Department of Land Resources and Environmental Sciences, Montana State University, Bozeman, MT, United States of America; 2 Department of Plant Sciences and Plant Pathology, Montana State University, Bozeman, MT, United States of America; 3 School of Natural Resources, University of Nebraska—Lincoln, Lincoln, NE, United States of America

**Keywords:** Antixenosis, Integrated pest management, Plant breeding, Insect resistance, Antibiosis

## Abstract

Plant resistance against insect herbivory has greatly focused on antibiosis, whereby the plant has a deleterious effect on the herbivore, and antixenosis, whereby the plant is able to direct the herbivore away from it. Although these two types of resistance may reduce injury and yield loss, they can produce selection pressures on insect herbivores that lead to pest resistance. Tolerance, on the other hand, is a more sustainable pest management strategy because it involves only a plant response and therefore does not cause evolution of resistance in target pest populations. Despite its attractive attributes, tolerance has been poorly studied and understood. In this critical, interpretive review, we discuss tolerance to insect herbivory and the biological and socioeconomic factors that have limited its use in plant resistance and integrated pest management. First, tolerance is difficult to identify, and the mechanisms conferring it are poorly understood. Second, the genetics of tolerance are mostly unknown. Third, several obstacles hinder the establishment of high-throughput phenotyping methods for large-scale screening of tolerance. Fourth, tolerance has received little attention from entomologists because, for most, their primary interest, research training, and funding opportunities are in mechanisms which affect pest biology, not plant biology. Fifth, the efforts of plant resistance are directed at controlling pest populations rather than managing plant stress. We conclude this paper by discussing future research and development activities.

## Introduction

Is tolerance the forgotten child of plant resistance? Its attributes are so appealing, yet it has received the least attention of the three types of plant resistance. As an insect pest management tactic, tolerance may be the consummate strategy (Pedigo & Higley, 1992). This is because a central tenet of integrated pest management (IPM) is that we tolerate some amount of pest injury. By making plants more tolerant of injury, we are achieving this important goal. Another goal is to use tactics that impose little selection pressure that will lead to pest resistance to those tactics. Contrary to antixenosis and antibiosis, tolerance does not affect insect biology or behavior (Smith, 2005); therefore, pests cannot become resistant to tolerant plants. Clearly, the conceptual advantages of tolerance in plant resistance cannot be discounted.

We believe there are several reasons why tolerance has not been developed as successfully as antibiosis and antixenosis. First, tolerance is difficult to identify and the mechanisms conferring it are poorly understood. Second, the genetics of tolerance are mostly unknown. Third, several obstacles still hinder the establishment of high-throughput phenotyping methods for large-scale screening of tolerance. Fourth, tolerance has received little attention from entomologists because, for most, their primary interest, research training, and funding opportunities are in mechanisms which affect pest biology, not plant biology. Fifth, the efforts of plant resistance are still directed at controlling pest populations rather than managing plant stress. In this paper, we discuss tolerance and the factors that have limited its use in plant resistance and IPM.

## Survey Methodology

Primary and secondary literature relevant to the topic of this paper was assessed using Web of Science (Clarivate Analytics) and Google Scholar. Key words such as “plant tolerance,” “host plant resistance,” “plant resistance,” “insect resistance,” “plant breeding,” “pest resistance,” “antibiosis,” and “antixenosis” were searched between 1 January and 31 May, 2017.

## Definitions and Concepts

Before discussing the five factors above in detail, we first need to define tolerance. In this instance, precisely defining terms is important because there continues to be considerable overlap in plant resistance definitions. At the outset, we recognize tolerance as distinctly different from the two other resistance types: antibiosis and antixenosis.

Antibiosis is a type of resistance that contains at least one plant characteristic that affects pest biology in a deleterious manner. Antixenosis is a type of resistance that contains at least one plant characteristic that directs a pest away from it. Tolerance is a type of resistance that causes the plant to compensate for pest injury to a degree exceeding non-tolerant plants (Kogan & Ortman, 1978; Painter, 1951; Smith, 2005). In an evolutionary context, tolerance is defined as the slope of the line describing the association between fitness and level of damage for a set of genetically related plants (Strauss & Agrawal, 1999). In agronomic situations, tolerant crop varieties are able to withstand injury and produce acceptable yields (Flinn et al., 2001; Qiu et al., 2011; Webster, 1990; Webster, Baker & Porter, 1991). From an ecological perspective, tolerant plants can maintain fitness in response to pest injury (Núñez Farfán, Fornoni & Valverde, 2007; Rosenthal & Kotanen, 1994).

Both antibiosis and antixenosis involve a plant response and a pest response. However, in the case of tolerance only a plant response is involved. Therefore, there is a nonreciprocal process associated with tolerance (Smith, 2005). This non-reciprocity has important ramifications when considering the use of tolerant cultivars in IPM programs.

Like antibiosis and antixenosis, tolerance is a type of resistance. Tolerance (as well as antibiosis and antixenosis) is not a mechanism of resistance (Smith, 1997). There are numerous mechanisms conferring tolerance (Koch et al., 2016; Strauss & Agrawal, 1999; Tiffin, 2000), just as there are numerous mechanisms for antibiosis and antixenosis (Du et al., 2009; War et al., 2012). Therefore, different and distinct mechanisms that enhance pest mortality collectively belong to the antibiosis resistance type.

What do we mean by stating that tolerant hosts can compensate for injury better than non-tolerant hosts? Plant response to biotic injury depends on four factors: the intensity of injury, the time of injury, the type of injury, the plant part injured, and interactions with environmental factors (Peterson & Higley, 2001). The intensity of injury is very important when considering the potential impact of the stressor on host yield or fitness. The relationship was described in the form of a damage curve by Tammes (1961), and has since been supported by substantial empirical evidence (Shelton, Hoy & Baker, 1990).

**Figure 1:**
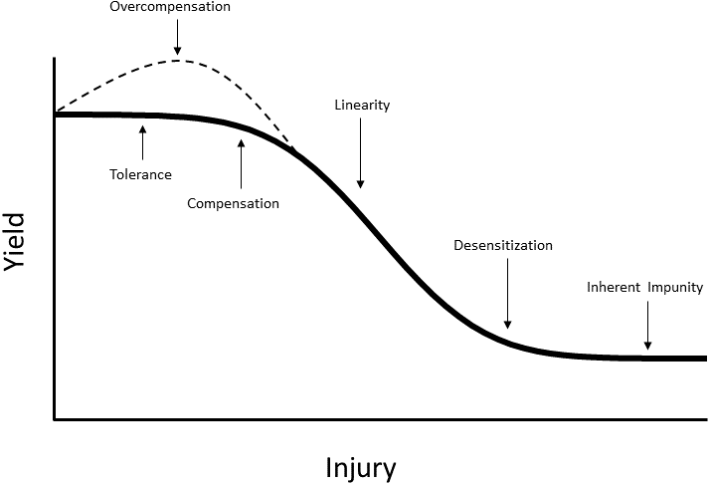
The damage curve relating intensity of injury to yield.

**Figure 2:**
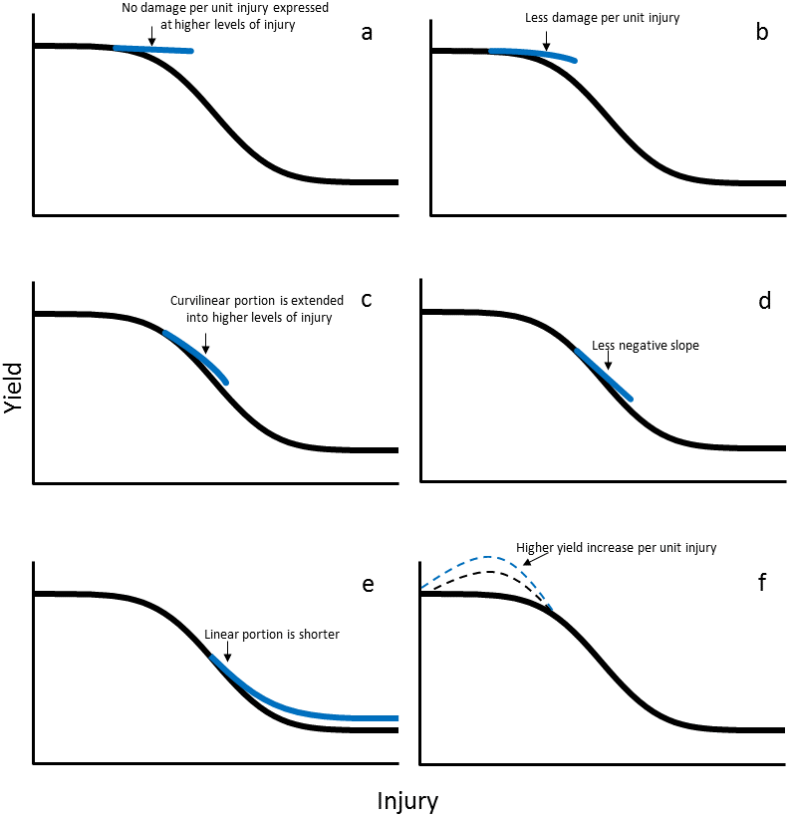
The damage curve showing different portions where tolerance can be expressed. (A) shows extending the initial zero slope of the damage curve, i.e., no damage per unit injury may be expressed at higher levels of injury for tolerant plants than for nontolerant plants; (B) shows that because this area is curvilinear (with a negative decreasing slope), tolerant plants may express less damage per unit injury; (C) shows that the curvilinear portion may be extended into higher levels of injury; (D) shows that the constant, negative slope (constant damage per unit injury) may have a less negative slope for tolerant plants; (E) shows that the linear portion may be shorter; (D) shows that desensitization and inherent impunity may occur at a higher yield; (F) shows that overcompensation (increasing yield per unit injury), may be expressed by both tolerant plants and nontolerant plants, but tolerant plants may express a higher yield increase per unit injury.

Pedigo, Hutchins & Higley (1986) defined portions of the damage curve more than two decades after its inception (Fig. 1). The damage curve can be used to present some of the basic aspects of tolerance. Although the initial portion of the damage curve is termed the tolerant region, there are actually four portions that can theoretically be expressed differentially by tolerant plants when compared with nontolerant plants. The damage curve can be altered by extending the initial zero slope of the damage curve; i.e., no damage per unit injury is expressed at higher levels of injury for tolerant plants than for nontolerant plants (Fig. 2A). Tolerant plants also may be able to affect the compensation area of the damage curve in two ways. First, because this area is curvilinear (with a negative decreasing slope), tolerant plants may express less damage per unit injury (Fig. 2B). Second, the slope is not altered, but the curvilinear portion is extended into higher levels of injury (Fig. 2C). The linear portion can also be affected by tolerant plants in two ways. First, the constant, negative slope (constant damage per unit injury) may have a less negative slope for tolerant plants (Fig. 2D). Second, the linear portion may be shorter. Therefore, desensitization and inherent impunity would occur at a higher yield (Fig. 2E). The last portion, overcompensation (increasing yield per unit injury), can be expressed by both tolerant plants and nontolerant plants; however, tolerant plants may express a higher yield increase per unit injury (Fig. 2F).

As we have suggested, the damage curve theoretically can be altered by plants expressing tolerance. The challenge remains to empirically identify empirically the portion or portions of the damage curve where tolerance is expressed by plants. In addition, simply because portions are identified in which tolerance is expressed does not mean those portions would be practical targets for plant breeding. The tolerance, overcompensation, and compensation portions (Figs. 2A, 2B and 2F) most likely would be the most practical, producer accepted, and economic targets for enhancing tolerance. Enhancing tolerance in the linearity, desensitization, and inherent impunity portions (Figs. 2C–2E) most likely would not be acceptable to producers because economic yield loss would already be occurring in these portions, except perhaps for lower injury areas of the linearity portion.

**Figure 3:**
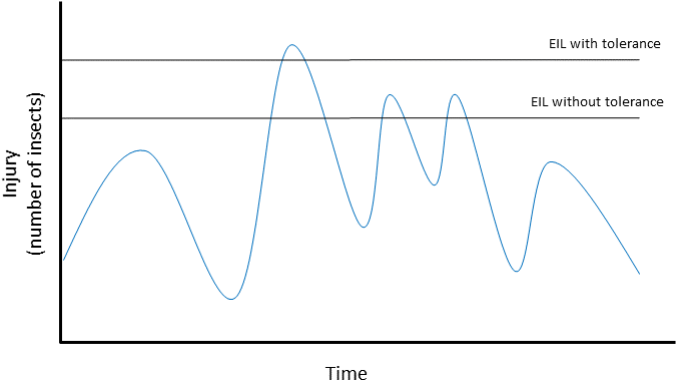
The relationship between injury (often expressed as number of insects), time, and the economic injury level with and without tolerance.

Tolerance can also be expressed in the context of economic injury level (EIL) parameters. The relationship between damage per unit injury and the EIL typically takes the form of Fig. 3. Because a tolerant plant ultimately expresses less damage per unit injury, the EIL will be greater for most levels of injury. This relationship can also be expressed when considering pest population levels over time and the EIL (Fig. 3).

## Constraints on the Development and use of Tolerance

### Identifying tolerance and characterizing tolerance mechanisms is difficult

A major factor contributing to the predominance of the use of antibiosis and antixenosis in plant resistance is sheer amenability. Antibiosis mechanisms often have been relatively easy to identify and breed for, mainly because they are, in many cases, determined by a single gene or major quantitative trait locus (QTL) and because their effects on herbivorous arthropods are readily apparent. We realize that the precise biochemical mechanisms for antibiosis in many systems are not known. For example, larval survival of the wheat stem sawfly, *Cephus cinctus*, is reduced by QTL on wheat chromosomes 2A, 3A, and 5B (Varella et al., 2015). Although specific mechanisms causing larval mortality have yet to be determined, this constraint has not hindered the identification of antibiosis and the ability to breed for wheat resistance to this pest.

Although antixenosis mechanisms are not as readily identifiable as antibiosis mechanisms, they still are more apparent than tolerance mechanisms. This is because antixenotic mechanisms usually involve plant morphological features that can be visually identified and because insect responses can be typically observed and measured. For example, the frego bract character in cotton and glandular trichomes in alfalfa (both of which discourage larval feeding and oviposition) are very apparent and efficacious (Jenkins & Parrott, 1971; Ranger & Hower, 2001). Even less visually apparent mechanisms such as surface waxes, tissue thickness, and chemical deterrents can be readily identified and assayed (Chamarthi et al., 2011; Jindal & Dhaliwal, 2011; Weaver et al., 2009).

In contrast to antixenosis and antibiosis, relatively little is known about tolerance. Tolerance to arthropod injury has been identified in alfalfa, barley, rice, sorghum, maize, wheat, cotton, cowpea, okra, muskmelon, turnip, and tea (Velusamy & Heinrichs, 1986), northern red oak, Spanish cedar, *Brassica rapa*, tall fescue, and perennial ryegrass (Strauss & Agrawal, 1999), lentil, sugarcane, soybean, potato, switchgrass, and cacao (Koch et al., 2016), cassava, tomato, and strawberry (Byrne et al., 1982; Gilbert, Chinn & Tanaka, 1966; Schuster et al., 1980). In some of these commodities, tolerance is a very important resistance attribute. For example, the resistance of sorghum to greenbug, *Schizaphis graminum*, is dependent on the survival of seedlings in response to feeding injury. This is clearly a tolerance response because resistant cultivars have no effect on greenbug biology or behavior (Schuster & Starks, 1973). In barley, the identification of Russian wheat aphid, *Diuraphis noxia*, populations virulent to resistance genes has prompted the development of tolerant cultivars (e.g., “Sydney” and “Stoneham”) in an attempt to reduce selection pressure on the aphid population, thus increasing the durability of genotypes (Haley et al., 2004; Marimuthu & Smith, 2012; Mornhinweg et al., 2009; Mornhinweg et al., 2012). Despite its successful use in some crops, little is known about the mechanisms underlying tolerance.

Tolerance is currently believed to be caused by six general physiological mechanisms: (i) increased net photosynthetic rate after herbivory, (ii) high relative growth rates, (iii) increased branching or tillering, (iv) pre-existing high levels of carbon storage in roots, (v) increased resource allocation from root to shoot after damage (Strauss & Agrawal, 1999), and (vi) up-regulation of detoxification mechanisms to counteract deleterious effects of herbivory (Koch et al., 2016). Possible morphological features of tolerance include protected meristems, number of meristems, and developmental plasticity (Rosenthal & Kotanen, 1994). At the molecular level, only a few transcripts (e.g., SNF1-related kinases, peroxidases, and catalases) have been identified as been involved in tolerance to herbivory through resource allocation (Schwachtje et al., 2006) or reactive oxygen species (ROS) detoxification mechanisms (Ramm et al., 2013; Smith et al., 2010).

It is important to note that mechanisms that contribute to tolerance may vary with herbivore specialization (e.g., specialists, generalists) (Agrawal & Fishbein, 2006; Delaney et al., 2008; Delaney et al., 2009; Delaney & Higley, 2006; Foyer, Verrall & Hancock, 2015), feeding guild (e.g., chewing, sucking) (Zhou et al., 2015), the plant’s symbiotic relationships (e.g., several milkweed species show increased tolerance to herbivory when associated with arbuscular mycorrhizal fungi) (Tao et al., 2016) and environmental conditions (Wise & Abrahamson, 2007). All of these factors complicate the identification and characterization of tolerance mechanisms. Also, some mechanisms are constitutively expressed while others are induced. Evaluation of germplasm showing induced tolerance must be done in the presence of pest populations, which is often more challenging due to seasonal variation in pest infestation at any given location.

Many crop varieties expressing tolerance have been discovered fortuitously. Development of resistant cultivars usually has been the result of general screening for any expression of resistance. For example, the development of the alfalfa cultivar “Team,” which is tolerant to alfalfa weevil, *Hypera postica*, injury, was the result of large-scale screenings of germplasm, in which more than two million seedlings were exposed to weevil infestation in an attempt to identify any resistance. After 10 years of breeding, “Team” was released in 1970. The cultivar is believed to express all three resistance types, but tolerance seems to be the dominant resistance factor (Barnes et al., 1970). It should be noted that the goal of the researchers was not to characterize mechanisms, but rather to produce a resistant variety. Large scale screenings focusing exclusively on plant tolerance have also been successful (Dunn et al., 2011).

### The genetics of tolerance are mostly unknown

The ability to predict phenotypic characteristics based on plant genotype is key to expediting the development of improved crops, mainly because it adds efficiency and precision to germplasm screening and selection. Nevertheless, understanding the genetics of plant tolerance to herbivory, as with any other trait, requires both the capability to detect polymorphic alleles and the recombination or segregation of these alleles.

To meet these requirements, large breeding populations need to be developed and screened. Lack of knowledge of the mechanisms underlying tolerance hinders the ability to precisely phenotype plants and interferes with the capacity of detecting polymorphisms. Despite the challenges, genetic variation in tolerance to herbivory has been demonstrated in crop and non-crop species (Marimuthu & Smith, 2012; Punnuri et al., 2013; Shen & Bach, 1997). Similar to antibiosis and antixenosis, tolerance seems to be mostly controlled by multiple loci and their interactions. Though QTL associated with tolerance to herbivory have been identified, to our knowledge, no gene has been cloned. Thus, further research should aim to enhance the genetic resolution of target QTL, which ultimately may result in the identification and cloning of causal genes.

### Establishing high-throughput screening methods for large-scale phenotyping of tolerance is difficult

One of the bottlenecks of breeding for insect tolerance is the difficulty in identifying diagnostic traits that can be easily, precisely, and consistently quantified under natural and/or imposed insect pressure. Screening methods that are laborious or time-consuming might be adequate for research purposes, but are for the most part not useful for screening the large number of lines regularly phenotyped in plant breeding programs.

For example, wheat tolerance to the bird cherry-oat aphid, *Rhopalosiphum padi*, can be assessed using a diverse set of methods that target a variety of plant traits (e.g., gain yield, thousand kernel mass, biomass ratios, and development of roots and shoots) (Dunn et al., 2011; Lamb & MacKay, 1995; Papp & Mesterházy, 1993). However, not all methods allow for the evaluation of thousands of plants in a timely manner (Dunn et al., 2011). Thus, the establishment of high-throughput phenotyping methods that allow for the precise characterization of a large number of lines will greatly contribute for the development of tolerant crop plants. Challenges associated with implementing high-throughput phenotyping for plant breeding programs are associated with costs of equipment, facilities, and software licenses (required for data analysis), lack of personnel trained for manipulation of large data sets, and lack of standards for experimental design and data analysis (Goggin, Lorence & Topp, 2015).

### Entomologists lack substantial training in plant biology

Because entomologists have been the primary participants in research on plant resistance to insects, entomocentric views have prevailed. Consequently, instead of concentrating on plant responses to insect-induced injury, entomologists have often used the plant to deliver a control tactic. This strategy reflects an inherent disadvantage in research training specialization (overspecialization?) of contemporary scientists (Jacobs & Frickel, 2009; Rhoten, 2004; Welter, 1989). Very few entomologists have had formal training in aspects of plant biology, such as photosynthesis, metabolism, anatomy, and water relations. Entomologists trained to consider the plant in insect-plant interactions potentially would improve research and development of tolerant cultivars. Additionally, interdisciplinary research teams may be able to develop tolerant cultivars. However, interdisciplinary research incorporating aspects of pest biology, plant physiology, and agronomy is still in its infancy (Peterson, 2001; Peterson & Higley, 2001).

### Plant resistance efforts are targeted toward the control of pest populations

We believe that plant resistance research, although overtly progressive and consistent with IPM, has largely followed a unilateral approach to pest management, similar to the control tactic of insecticide use common in the 1950s and early 1960s. Through antixenosis, and especially antibiosis mechanisms, resistant cultivars essentially are suppressing insect populations. Unlike insecticide use, the adverse environmental impacts of using resistant cultivars are quite low. In this respect, resistant cultivars satisfy one objective of IPM: minimizing detrimental environmental effects. However, cultivars with antibiotic mechanisms place selection pressure on insect populations, potentially encouraging the development of resistance. Although, resistant cultivars may represent a more desirable control tactic, they do not necessarily represent a truly sustainable pest management practice. New approaches for incorporating resistance in plants also will not be sustainable. For example, plants that are engineered to produce the *Bacillus thuringiensis* (Bt) toxin have selected for resistance (even when pest populations were not economic) (Tabashnik et al., 2008).

The issue of control versus management in IPM is a critical factor when attempting to understand why tolerance is not as prominent in plant resistance. The terms “control” and “management” as they relate to pest management have been discussed (Higley & Pedigo, 1993; Higley & Pedigo, 1996; Menalled et al., 2016; Pedigo & Higley, 1996; Pedigo & Rice, 2009). Briefly, “control” implies a program focused on the pests themselves, and, in particular killing pests. Therefore, this often results in strong selection pressure for resistance. The focus on killing pests includes the highly efficacious antibiotic tactic represented by Bt crops. In contrast, “management” implies a program focused on the “judicious use of means to accomplish a desired end” (Pedigo & Higley, 1996). Tolerance, then, as a type of plant resistance, clearly fits well with management.

### Other biological factors

Conceptually, tolerance has very attractive attributes for use in IPM programs. However, because tolerance has been so poorly studied and understood, we do not know if or how much specific interactions with the environment (such as drought or heat stress) will render the tolerant variety completely susceptible to pest injury. This is especially relevant in the face of climate change and the increase in drought-prone areas. In non-crop species for instance, drought has been shown to limit a plant’s ability to tolerate herbivory (Atala & Gianoli, 2009; Gonzáles, Suárez & Molina-Montenegro, 2008). But even closely related species of plants may respond differently to herbivory under drought conditions (Shibel & Heard, 2016). Thus, the impact of environment on the plant’s ability to tolerate insect herbivory might have to be assessed for each crop species and/or variety.

In several crop systems, some arthropod species move from one crop species to another during their life cycle. For example, in North Carolina the corn earworm, *Helicoverpa zea*, may injure corn, tobacco, wild hosts, soybean, and cotton. Having just one crop species in an area tolerant to corn earworm injury may result in unacceptable populations for the other crop species.

### Socioeconomic factors

In the US, growers attempt to control pests to avoid risk as much as, if not more, than to optimize yields (Higley, 2006). Understandably, then, growers may be uncomfortable with a large number of pests feeding on their tolerant cultivar. In this case, the cultivar may be able to tolerate the injury, but the grower cannot. The attitude that the “only good bug is a dead bug” is undoubtedly alive and well in modern farming systems. Additionally, private companies may not embrace tolerant cultivars because they do not want their customers to doubt that their varieties are indeed resistant. Therefore, education about tolerance and tolerant cultivars must be a priority if this resistance strategy is to be successful.

Tolerant cultivars must be agronomically desirable. Nguessan & Quisenberry (1994) identified several rice lines that are tolerant to rice weevil, *Sitophilus oryzae*, injury. However, they were not agronomically desirable. This is a major limitation to incorporating tolerance into crops and must be addressed by researchers. Another major limitation is that tolerant crops may be more vulnerable to cosmetic damage than crops displaying other types of resistance. Consumer preference for fruits and vegetables, for example, is influenced by product appearance. Thus consumer preference for undamaged food products might limit the use of tolerance in some crop species.

## Conclusions and Recommendations

Although antixenosis and antibiosis may lessen or negate the need for pesticides applied to the crop, they can produce selective pressures on insect populations that are similar to pesticides. The management tactic may be more environmentally acceptable and therefore may be more popular with policy makers and the public, but if sufficient selective pressure is placed on the pest population the tactic is not sustainable in the long term (Kennedy et al., 1987; Tolmay, Lindeque & Prinsloo, 2007). Tolerance, as a resistance mechanism, is very appealing because it is a sustainable tactic (Kennedy et al., 1987; Pedigo, 1995; Pedigo & Rice, 2009; Rausher, 2001). By not placing selective pressure on insect populations, it essentially factors the pest out of the equation. Additionally, EILs for tolerant varieties would be substantially higher than for susceptible varieties. Therefore, reduced pesticide inputs would result. Because of these factors, tolerance is a more stabilizing management strategy for pests.

Velusamy & Heinrichs (1986) list three factors they believe are responsible for the lack of attention to tolerance. They include: a lack of suitable techniques to identify and incorporate tolerance into crops; the ability of tolerant cultivars to serve as reservoirs for insect vectors of viruses; and, the lack of basic information on the inheritance of tolerance. We believe they have identified three factors that potentially constrain the development of tolerance. However, we believe our five factors are more encompassing, reflecting the biological, economic, and social constraints on tolerance development. For example, the lack of suitable techniques to identify tolerance is really a reflection of the lack of understanding about basic physiological mechanisms underlying tolerance.

Before substantial work on tolerance development can occur, we must conduct basic research on the physiological and biochemical mechanisms of tolerance. This must involve interdisciplinary research between plant scientists and entomologists. Beyond an interdisciplinary focus, it is important that there is awareness from applied researchers about research and findings from fundamental researchers and vice-versa. There are longstanding issues of lack of communication between biologists, ecologists, and agricultural scientists (Higley, Browde & Higley, 1993) and this must be addressed before tolerance can be appreciably advanced.

More generally, research on the physiological responses of plants to arthropod injury (irrespective of tolerance) must progress beyond what is currently known. Higley, Browde & Higley (1993) argued that a focus on plant physiology provides a common language for characterizing plant stress and is essential for integrating understanding of stress. Peterson & Higley (1993) and Peterson (2001) discuss approaches for synthesizing plant responses to arthropod injury.

Based on the factors we have discussed above, we believe the development and use of tolerance in plant resistance to arthropods can be hastened by achieving the following goals: (1) research characterizing the physiological mechanisms underlying tolerance; (2) research determining the physiological responses of plants to arthropod injury; (3) encouragement of interdisciplinary research and communication among entomologists, plant scientists, ecologists, and molecular biologists; and (4) progression of IPM theory to a true paradigm for managing plant stress. Ultimately, to understand the conceptual importance of tolerance to plant resistance, the importance of tolerance to IPM must be appreciated.

## Additional Information and Declarations

### Data Availability

The following information was supplied regarding data availability: This is a review article which did not generate raw data.
